# Supplementary material for: An intrinsically disordered region mediates RNA-binding selectivity and cellular activities of LARP6
Source: Nat Commun. 2026 Feb 19;17:2939. doi: 10.1038/s41467-026-69789-z (PMC13031558; doi:10.1038/s41467-026-69789-z)

Capraro et al., Supplementary Information

Supplementary Tables:

Supplementary Table 1: List of all RNA and DNA oligonucleotides used in this study.

Oligo ID	Sequence (5'-3')	Company
**siRNAs**		
Custom LARP6 siRNA	GGGAGAAAGGCCUGGAAUUUU	Horizon Discovery
ON-TARGETplus Non-targeting Control Pool (scramble)	UGGUUUACAUGUCGACUAA, UGGUUUACAUGUUGUGUGA, UGGUUUACAUGUUUUCUGA, UGGUUUACAUGUUUUCCUA	Horizon Discovery
**iCLIP L3-NNN-App adapters**		
L3-ATT-App	/5Phos/WN ATT AGA TCG GAA GAG CGG TTC AG/3Bio/	IDT
L3-AGG-App	/5Phos/WN AGG AGA TCG GAA GAG CGG TTC AG/3Bio/	IDT
L3-TTA-App	/5Phos/WN TTA AGA TCG GAA GAG CGG TTC AG/3Bio/	IDT
L3-TGC-App	/5Phos/WN TGC AGA TCG GAA GAG CGG TTC AG/3Bio/	IDT
L3-CTG-App	/5Phos/WN CTG AGA TCG GAA GAG CGG TTC AG/3Bio/	IDT
L3-CGT-App	/5Phos/WN CGT AGA TCG GAA GAG CGG TTC AG/3Bio/	IDT
**iCLIP reverse transcription primers**		
irCLIP_ddRT_12	/5Phos/ WWW GTGGA NNNN AGATCGGAAGAGCGTCGTGAT /iSp18/ GGATCC /iSp18/ TACTGAACCGC	IDT
irCLIP_ddRT_13	/5Phos/ WWW TCCGG NNNN AGATCGGAAGAGCGTCGTGAT /iSp18/ GGATCC /iSp18/ TACTGAACCGC	IDT
irCLIP_ddRT_14	/5Phos/ WWW TGCCT NNNN AGATCGGAAGAGCGTCGTGAT /iSp18/ GGATCC /iSp18/ TACTGAACCGC	IDT
**iCLIP PCR primers**		
P3 Solexa	CAAGCAGAAGACGGCATACGAGATCGGTCTC GGCATTCCTGCTGAACCGCTCTTCCGATCT	IDT
P5 Solexa	AATGATACGGCGACCACCGAGATCTACACTCT TTCCCTACACGACGCTCTTCCGATCT	IDT
**5' biotinylated RNA oligos for BLI**		
Peak 1 (P1)	CUAAAUACAACACUGAUACUAGAUU	Horizon Discovery
Peak 2 (P2)	GGAAAUGGGCAGACUGAACCAGUCC	Horizon Discovery
Upstream 1 (U1)	ACUGUUCGUCACUCAAAUGAAUUUG	Horizon Discovery
Upstream 2 (U2)	CCCUCGGGGCUCCUGAAUAUCAGUC	Horizon Discovery
Downstream 1 (D1)	AGGUGGUGAAUUUUCCAAGAACAUA	Horizon Discovery
Downstream 2 (D2)	GUUUAAGUUGAUUAAAAAUGCUUUU	Horizon Discovery
PolyC	CCCCCCCCCCCCCCCCCCCCCCCCC	Horizon Discovery
**5' 6-FAM (fluorescein) RNA oligo for MST**		
Peak 1 (P1)	CUAAAUACAACACUGAUACUAGAUU	Merck

Supplementary Table 2: Essential SAXS details on samples, data acquisition, analysis parameters, modelling fitting and software used.

	LARP6 La-module apo	LARP6 La-module/P1	LARP6 NTD apo	LARP6 NTD/P1	LARP6 NTR apo
**(*a*) Sample details**					
Organism	*H. sapiens*				
Source (recombinant expression)	*E. coli* Ros2™				
Description – sequence + bound ligands.	Protein sequence 1	Protein sequence 1, RNA sequence 1	Protein sequence 2	Protein sequence 2, RNA sequence 1	Protein sequence 3
Extinction coefficient	25440 $M^{-1}cm^{-1}$	287040 $M^{-1}cm^{-1}$	32430 $M^{-1}cm^{-1}$	294030 $M^{-1}cm^{-1}$	6990 $M^{-1}cm^{-1}$
Partial specific volume	0.70–0.75 mL/g (average of experimentally determined partial specific volumes for soluble, globular proteins)				
M from chemical composition	26883.17 Da	34802.03 Da	33917.62 Da	41836.48 Da	8727.11 Da
Loading volume	65 µL				
Flow rate	0.2 mL/min				
Concentration measured<br>(Adjusted by extinction coefficients in spectroscopy)	4–5 mg/mL	4–5 mg/mL<br>1:2 Protein:RNA molar ratio	3–4 mg/mL	3–4 mg/mL<br>1:2 Protein:RNA molar ratio	4–5 mg/mL
Solvent details	25 mM Tris-HCl, 100 mM KCl, 5 mM $MgCl_2$, 1 mM DTT, pH 7.5				
**(*b*) SAXS data collection parameters**					

Source, instrument and description	B21 beamline at Diamond Light Source[1]
Wavelength	0.9464 Å
Beam geometry (size, sample – detector distance)	The beam centres (X and Y) were located at 129.53 mm and 19.92 mm, respectively, with a size of 0.05×0.05 mm at the detector and 1×0.25 mm at the sample site.<br><br>Data collection was performed using an EigerX 4M detector (Dectris) placed 3702.5 mm from the sample.
q-measurement range	0.0045–0.34 $Å^{-1}$
Absolute scaling method	Absolute intensity scaled to water scatter at 0.0163
Method for monitoring radiation damage, X-ray dose where relevant	50 mM TRIS base, mitigation through continuous sample flow and data analysis assessment
Exposure time, number of exposures	A total of 600 frames were collected at a rate of one frame per second
Sample configuration including path length and flow rate where relevant	Path length: 1.5 mm, flow rate: 0.2 mL/min
Sample temperature	Samples were queued at 4°C and the experiments conducted at 15°C

(*c*) Software employed for SAXS data reduction, analysis and interpretation

SAXS data reduction to (sample – solvent), extrapolation, merging, desmearing etc. as relevant	ScÅtter IV
Calculation of extinction coefficient from sequence	ProtParam
Basic analyses: Guinier, *P*(*r*), Porod volume, volume of correlation	ScÅtter IV

(*d*) **Structural parameters**					
**Guinier Analysis**					
I(0)	1.2e-2	7.3e-2	3.7e-2	6.7e-2	1.3e-2
R_g	2.8 nm	3.0 nm	3.6 nm	3.7 nm	2.7 nm
qR_g range	0.31–1.61	0.33–1.43	0.35–1.49	0.29–1.39	0.289–1.491
Quality-of-fit parameter (R^2 of q^2 vs ln[I(q)])	0.9955	0.9973	0.9975	0.9872	0.9591
M as observed in SEC-MALLS	36 kDa	46 kDa	45 kDa	48 kDa	11 kDa
**P(*r*) analysis**					
I(0)	1.2e-2	7.3e-2	3.7e-2	6.7e-2	1.3e-2
R_g	2.8 nm	3.0 nm	3.6 nm	3.7 nm	2.7 nm
d_{max}	8.3 nm	12 nm	15 nm	15.3 nm	9.4 nm
q range ($Å^{-1}$)	0.009–0.185	0.011–0.184	0.010–0.147	0.019–0.182	0.017–0.216
Quality-of-fit parameter (χ^2)	1.12	1.16	1.31	1.10	1.03
V_P and/or V_c	59 nm^3	100 nm^3	138 nm^3	138 nm^3	21 nm^3
(*e*) **Data and model SasBDB deposition IDs**					
https://www.sasbdb.org/	SASDYW5	SASDYZ5	SASDYX5	SASDY26	SASDYY5
**(f) Sequences**					
**Protein sequence 1 (LARP6 La-module)**	GTTASGGENEREDLEQEWKPPDEELIKKLVDQIEFYFSDENLEKDAFLLKHVRRNKLGYVSVKL LTSFKKVKHLTRDWRTTAHALKYSVVLELNEDHRKVRRTTPVPLFPNENLPSKMLLVYDLYLSP				

	KLWALATPQKNGRVQEKVMEHLLKLFGTFGVISSVRILKPGRELPPDIRRISSRYSQVGTQECAI VEFEEVEAAIKAHEFMITESQGKENMKAVLIGMKPPKKKP
**Protein sequence 2 (LARP6 NTD)**	MAQSGGEARPGPKTAVQIRVAIQEAEDVDELEDEEEGAETRGAGDPARYLSPGWGSASEEEP SRGHSGTTASGGENEREDLEQEWKPPDEELIKKLVDQIEFYFSDENLEKDAFLLKHVRRNKLGY VSVKLLTSFKKVKHLTRDWRTTAHALKYSVVLELNEDHRKVRRTTPVPLFPNENLPSKMLLVYD LYLSPKLWALATPQKNGRVQEKVMEHLLKLFGTFGVISSVRILKPGRELPPDIRRISSRYSQVGT QECAIVEFEEVEAAIKAHEFMITESQGKENMKAVLIGMKPPKKKP
**Protein sequence 3 (LARP6 NTR)**	MAQSGGEARPGPKTAVQIRVAIQEAEDVDELEDEEEGAETRGAGDPARYLSPGWGSASEEEP SRGHSGTTASGGENEREDLEQ
**RNA sequence (CTNNA1 P1 RNA)**	5' CUAAAUACAACACUGAUACUAGAUU 3'

Supplementary Figures:

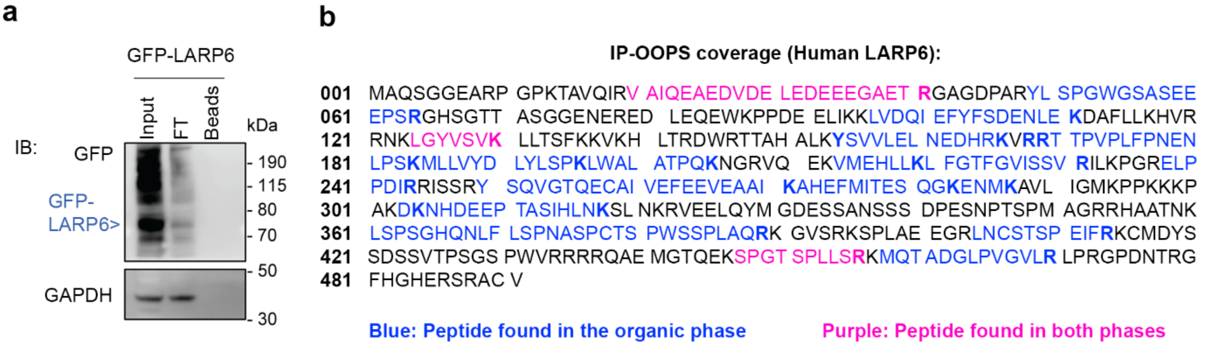

Supplementary Figure 1: Confirmation of GFP-LARP6 immunoprecipitation and peptide coverage for IP-OOPS analysis.

a) MDA-MB231 cells stably expressing GFP-LARP6 were subjected to UV-C crosslinking, followed by lysis and clearance of lysates with centrifugation. Aliquots were collected from the lysates before the IP (input), from the flowthrough (FT), and from the beads after the elution of GFP-LARP6 (beads) to validate the efficiency of the pulldown and elution. The expected band for GFP-LARP6 is indicated in blue. The smear above the GFP-LARP6 band corresponds to the RNA-crosslinked LARP6. Note: one representative experiment out of two independent repeats is shown.

b) Human LARP6 amino-acid sequence, with the MS-identified IP-OOPS peptides highlighted. Peptides detected only from non-crosslinked LARP6 that partitions to the organic phase are highlighted in blue; peptides also detected at the second OOPS interface (crosslink-adjacent) are highlighted in magenta. Tryptic cleavage sites are shown in bold.

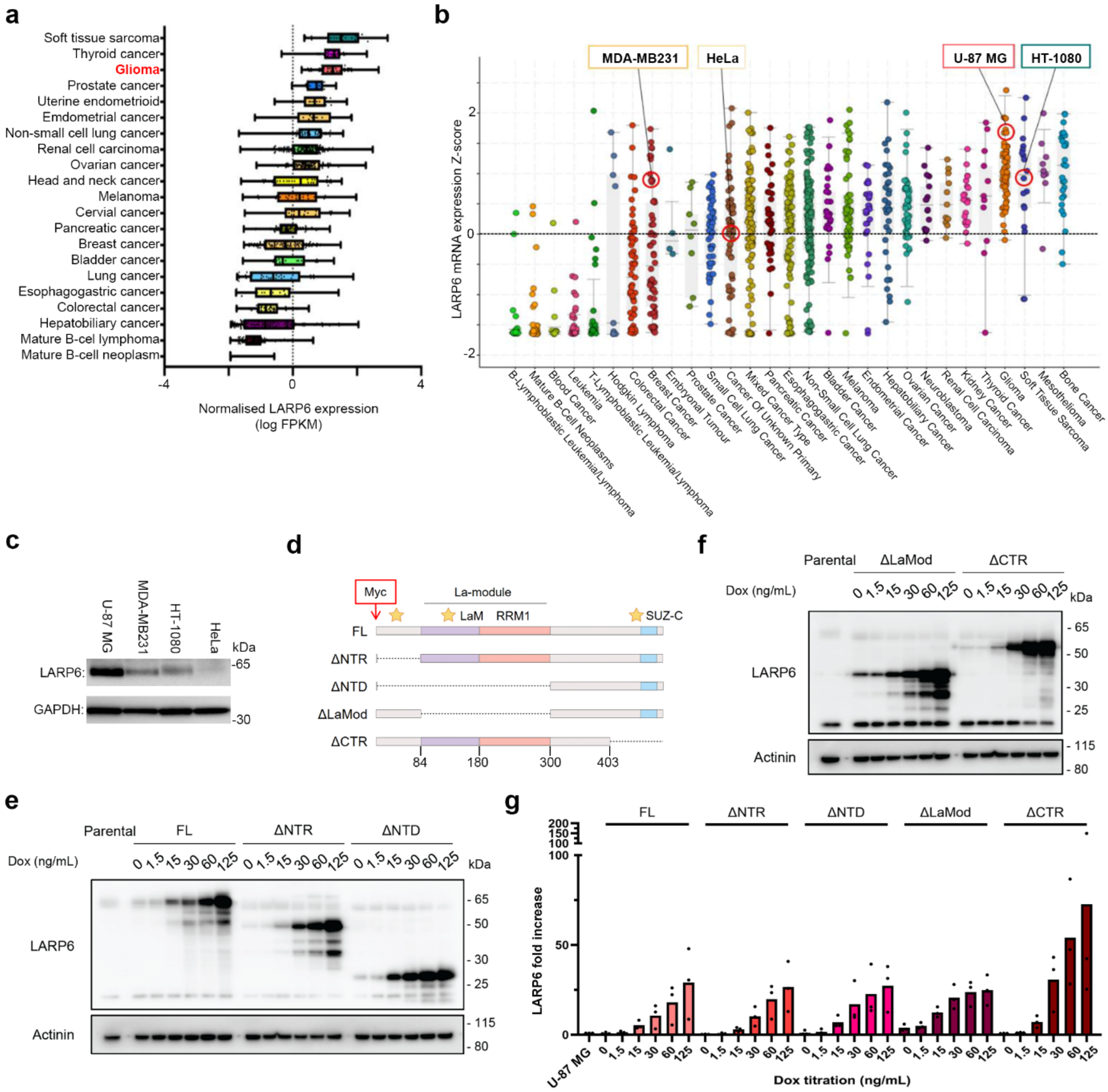

Supplementary Figure 2: A doxycycline inducible U-87 MG glioblastoma cell-line model for characterisation of different LARP6 RNA binding regions in cells.

a) LARP6 RNA expression levels across various cancer types from the TCGA database (https://www.cancer.gov/tcga) were extracted and exported from cBioPortal (cbioportal.org). Normalised LARP6 expression is displayed as log FPKM (fragment per kilobase of transcript per million). The boxplots mark the lower and upper quartiles, with the median marked by the vertical line. The whiskers extend to the minima and maxima.

b) LARP6 RNA expression values across various cancer cell lines. The expression data is from the Broad Institute Cancer Cell Line Encyclopaedia[2] and was exported from cBioPortal. Normalised LARP6 expression is displayed as z-scores relative to all samples in log RPKM (read per kilobase per million mapped reads). Cell lines are grouped according to their primary tumour site. The selected investigated cell lines are highlighted with red circles.

c) LARP6 protein expression levels across indicated cell lines. LARP6 protein expression was evaluated by Western blotting in the indicated cell lines of different cancer origins, chosen based on (b). Cancer origin: U-87 MG: glioblastoma; MDA-MB-231: triple negative breast adenocarcinoma; HT-1080: fibrosarcoma; HeLa: cervical cancer. Note: one representative experiment out of three independent repeats is shown.

d) Schematic representation of myc-LARP6 inducible deletion mutants expressed in U-87 MG glioblastoma cells. All mutants express a myc-tag at the N-terminus. The IP-OOPS identified RNA-binding regions are marked by stars.

e) Western blot analysis of FL, ΔNTR, and ΔNTD myc-LARP6 deletion mutants induction of expression with different doses of doxycycline. Each myc-LARP6 variant was induced with 0–125 ng/mL doxycycline for 24 hours, before lysis and Western blot analysis with the indicated antibodies. Note: one representative experiment out of three independent repeats is shown.

f) Western blot analysis of ΔLa-module, and ΔCTR myc-LARP6 deletion mutants induction of expression with different doses of doxycycline. Each myc-LARP6 variant was induced with 0–125 ng/mL doxycycline for 24 hours, before lysis and Western blot analysis with the indicated antibodies. Note: one representative experiment out of three independent repeats is shown.

g) Quantification of LARP6 expression levels from (e) and (f). LARP6 values were normalised to the endogenous LARP6 in parental U-87 MG cells (n = 3 biological replicates).

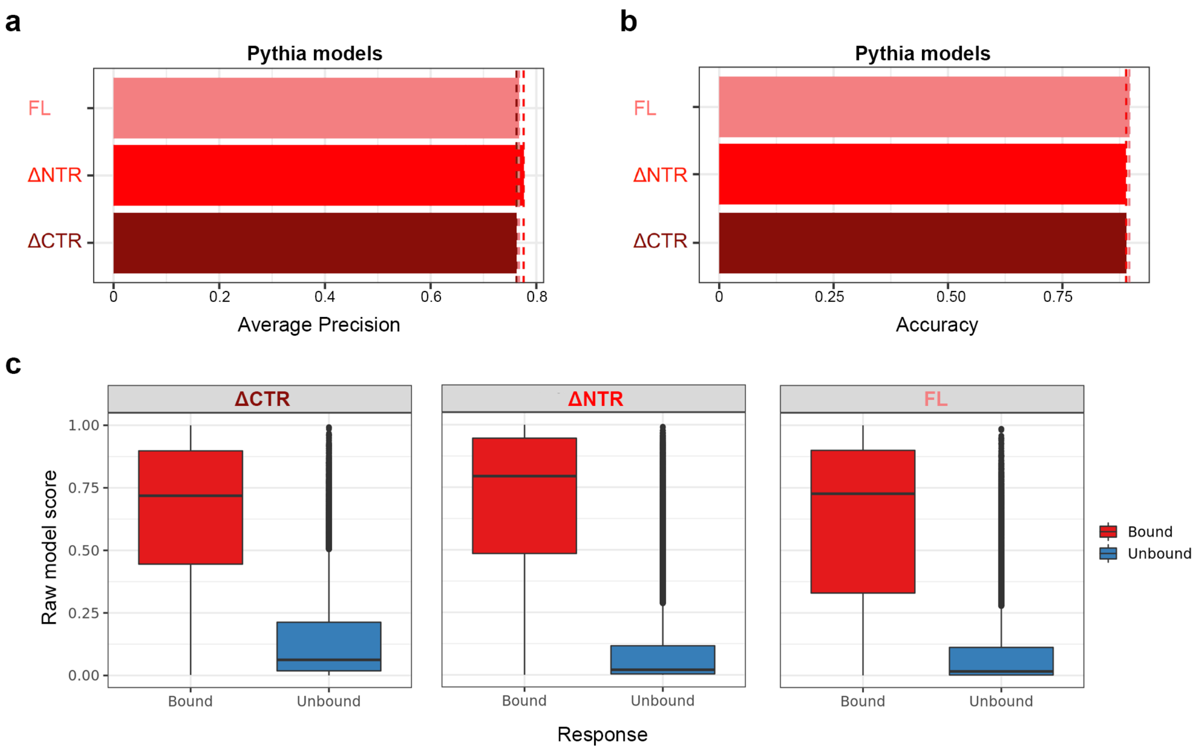

Supplementary Figure 3: Benchmarking of Pythia for analysis of LARP6 wild-type and mutant RNA binding profiles.

a) Average precision calculated for Pythia models trained on the FL, ΔNTR, and ΔCTR myc-LARP6 iCLIP peak datasets.

b) Average accuracy calculated for Pythia models trained on the FL, ΔNTR, and ΔCTR myc-LARP6 iCLIP peak datasets.

c) Raw model scores for predicting bound vs unbound peak fractions for Pythia models trained on the FL, ΔNTR, and ΔCTR myc-LARP6 iCLIP peak datasets. Th boxplots mark the lower and upper quartiles with the median marked by the horizontal line. The whiskers extend to the minima and maxima, excluding any outliers, which are individually plotted.

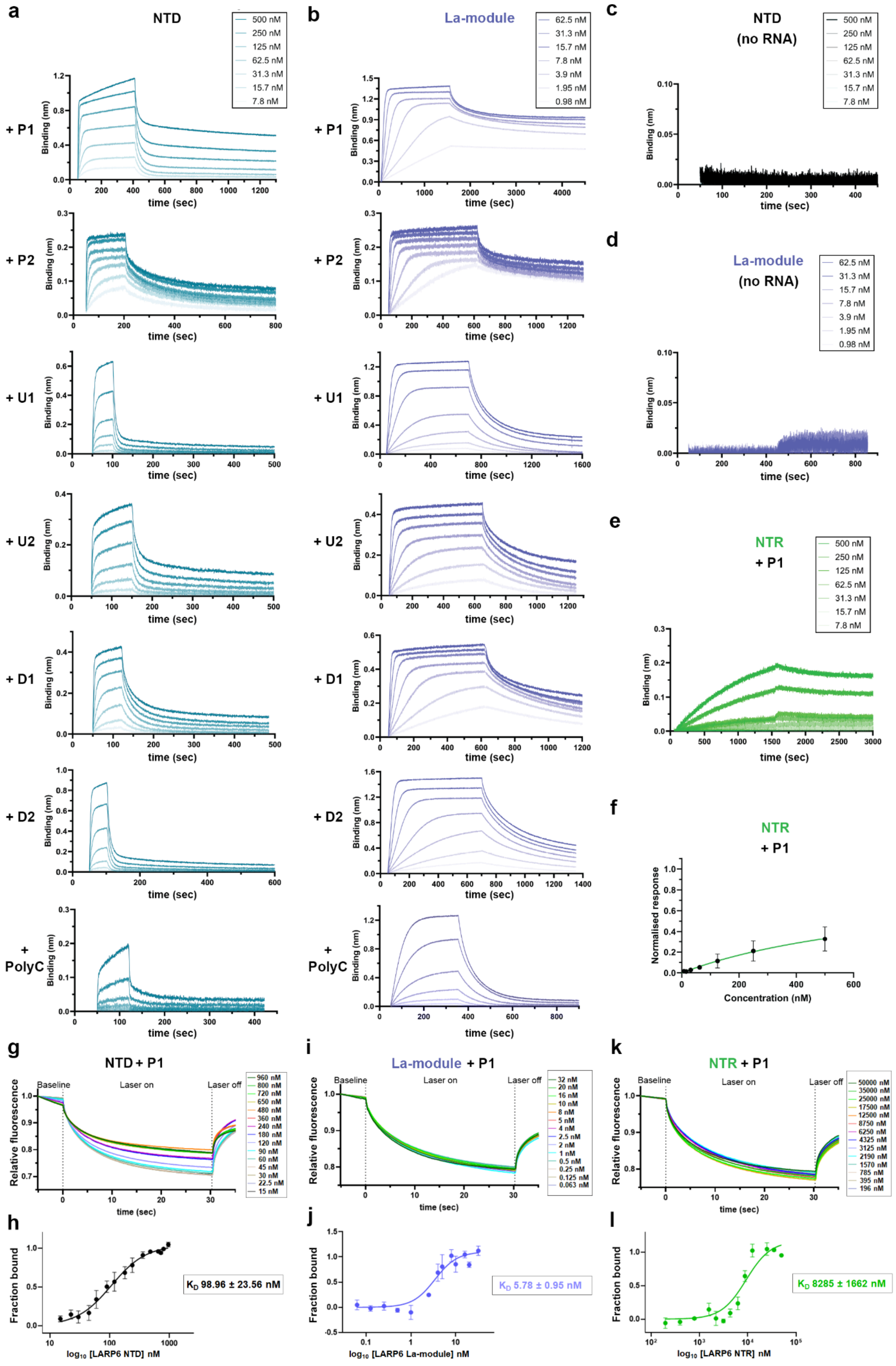
a
NTD
b
La-module
c
NTD
(no RNA)
d
La-module
(no RNA)
e
NTR
+ P1
f
NTR
+ P1
+ P1
+ P2
+ U1
+ U2
+ D1
+ D2
+ PolyC
Binding (nm)
time (sec)
Normalised response
Concentration (nM)
g
NTD + P1
i
La-module + P1
k
NTR + P1
Baseline
Laser on
Laser off
Relative fluorescence
h
j
l
Fraction bound
K_D 98.96 ± 23.56 nM
K_D 5.78 ± 0.95 nM
K_D 8285 ± 1662 nM
log₁₀ [LARP6 NTD] nM
log₁₀ [LARP6 La-module] nM
log₁₀ [LARP6 NTR] nM

Supplementary Figure 4: Analysis of the *in vitro* RNA binding activity of different LARP6 proteins.

a) Representative BLI association-dissociation profiles of the NTD segment of LARP6, against the indicated RNA oligonucleotides. The protein concentrations used for each plot are displayed within the top right box.

b) Representative BLI association-dissociation profiles of the La-module of LARP6, against the indicated RNA oligonucleotides. The protein concentrations used for each plot are displayed within the top right box.

c) BLI association-dissociation profile of the NTD of LARP6 in absence of RNA, as control for unspecific binding to empty sensors.

d) BLI association-dissociation profile of the La-module of LARP6 in absence of RNA, as control for unspecific binding to empty sensors.

e) Representative association-dissociation profile of BLI experiments performed with the NTR of LARP6 and the P1 CTNNA1 3'UTR RNA oligonucleotide.

f) Steady state BLI binding analysis of the NTR of LARP6 and the P1 CTNNA1 3'UTR RNA oligonucleotide. Error bars = mean ± SD (n = 3 independent replicates).

g) Representative normalised Microscale Thermophoresis (MST) time-traces of the NTD of LARP6 upon binding to 5' 6-FAM-labelled CTNNA1 P1 RNA.

h) MST-calculated binding curve, showing the fraction of NTD–bound P1 RNA as a function of protein concentration. A mid-nanomolar K_D value (99 nM) was estimated for the NTD interaction with P1 through curve fitting. Error bars = mean ± SEM (n = 4 independent replicates), which is in line with the BLI estimation (Table 1).

i) Representative normalised Microscale Thermophoresis (MST) time-traces of the La-module of LARP6 upon binding to 5' 6-FAM-labelled CTNNA1 P1 RNA.

j) MST-calculated binding curve, showing the fraction of La-module–bound P1 RNA as a function of protein concentration. A low-nanomolar K_D (5.8 nM) value was estimated for the NTD interaction with P1 through curve fitting. Error bars = mean ± SEM (n = 4 independent replicates), which is in line with the BLI estimation (Table 1).

k) Representative normalised Microscale Thermophoresis (MST) time-traces of the NTR of LARP6 upon binding to 5' 6-FAM-labelled CTNNA1 P1 RNA.

l) MST-derived binding curve, showing the fraction of NTR–bound P1 RNA as a function of protein concentration. A micromolar K_D (8.3 μM) value was estimated for the NTR interaction with P1 through curve fitting. Error bars = mean ± SEM (n = 4 independent replicates), which is in line with the BLI estimation (Table 1).

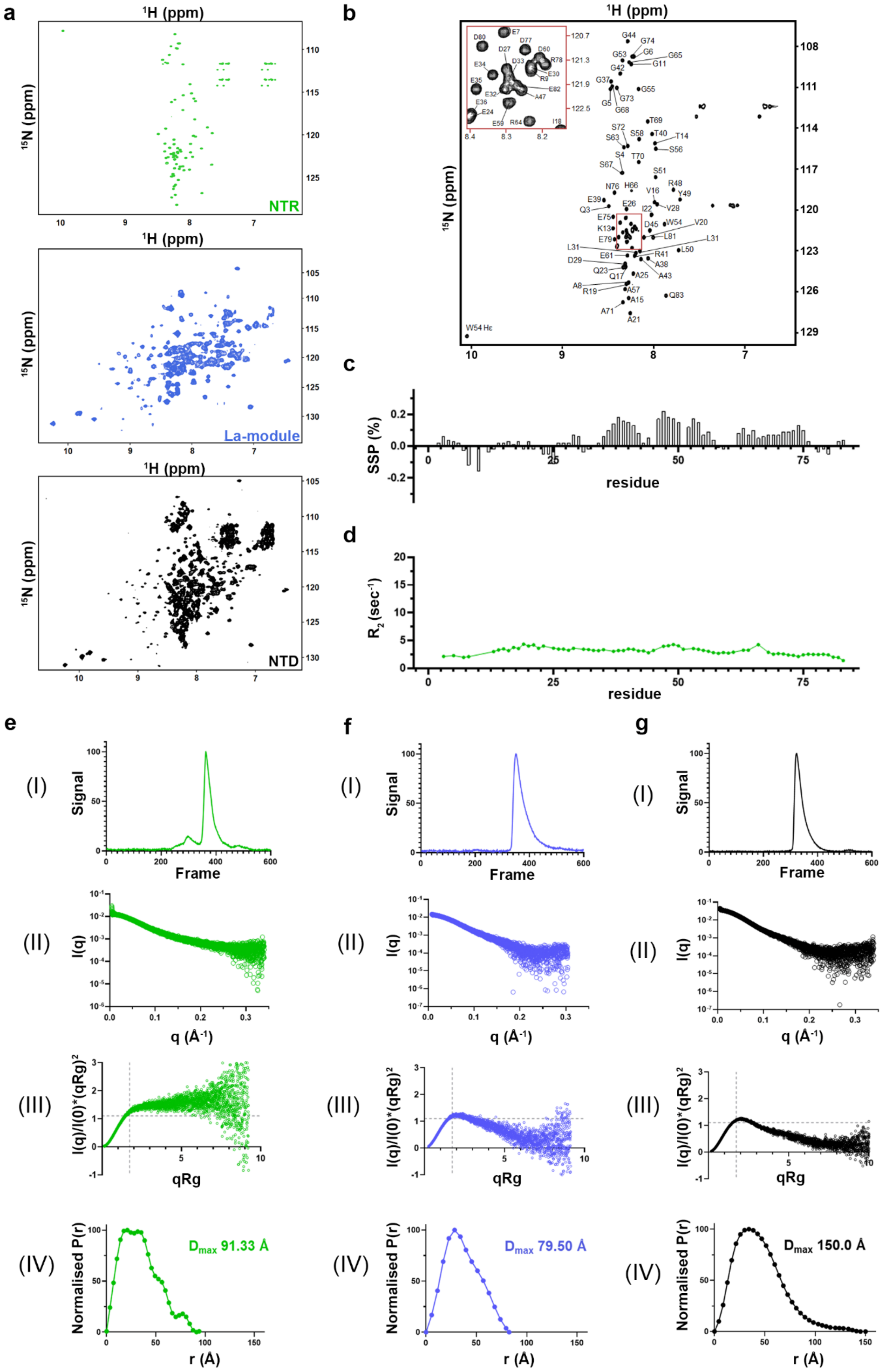
a
^{1}H (ppm)
^{15}N (ppm)
NTR
La-module
NTD
b
^{1}H (ppm)
^{15}N (ppm)
W54 Hε
c
SSP (%)
residue
d
R_2 (sec^{-1})
residue
e
f
g
(I)
Signal
Frame
(II)
I(q)
q (Å^{-1})
(III)
I(q)/I(0)*(qRg)^2
qRg
(IV)
Normalised P(r)
r (Å)
D_{max} 91.33 Å
D_{max} 79.50 Å
D_{max} 150.0 Å

Supplementary Figure 5: Biophysical characterisation of LARP6 NTR, La-module, and NTD segments in apo state.

a) Full ^{1}H-^{15}N TROSY NMR spectra of LARP6 NTR, La-module, and NTD (green, blue and black respectively).

b) ^{1}H-^{15}N TROSY NMR spectrum of LARP6 NTR indicating backbone amide resonances assignment. All resonances clustered in the region ranging between ~7.5 and 8.5 ppm, which is characteristic of intrinsic disordered regions. The assignment was performed on the NTR in isolation. The assigned amide groups are labelled. The red square zooms in a crowded region.

c) Secondary structure propensity analysis (SSP) of LARP6 NTR. SSPs were derived from chemical shift deviations of CO, Cα, Cβ, Hα from random coil values[3]. Positive and negative values indicate propensity towards α-helices and β-sheets, respectively. The overall low SSP scores (5% ≤ SSP ≤ 22%) is in agreement with lack of defined structure of the NTR of LARP6 in isolation.

d) NMR R_2 relaxation analysis of residues in the NTR in isolation, showing values consistent with intrinsic disorder across the entire region.

e) SEC-SAXS characterisation of LARP6 NTD. The normalised signal of the integral ratio to the background for each frame of SEC-SAXS (I), the X-ray scattering curve obtained after buffer normalisation and averaging (II), the dimensionless Kratky plot (III), and the P(r) distance distribution plot (IV) are depicted.

f) SEC-SAXS characterisation of LARP6 La-module. The normalised signal of the integral ratio to the background for each frame of SEC-SAXS (I), the X-ray scattering curve obtained after buffer normalisation and averaging (II), the dimensionless Kratky plot (III), and the P(r) distance distribution plot (IV) are depicted.

g) SEC-SAXS characterisation of LARP6 NTR. The normalised signal of the integral ratio to the background for each frame of SEC-SAXS (I), the X-ray scattering curve obtained after buffer normalisation and averaging (II), the dimensionless Kratky plot (III), and the P(r) distance distribution plot (IV) are depicted.

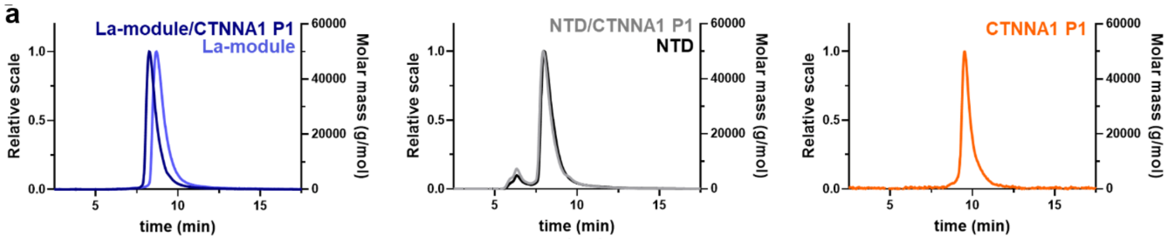

Construct	Expected MW	Observed MW
La-module	26.9 kDa	36.4 ± 2.4 kDa
La-module/CTNNA1 P1	34.8 kDa	46.3 ± 1.0 kDa
NTD	33.9 kDa	45.2 ± 0.5 kDa
NTD/CTNNA1 P1	41.8 kDa	47.9 ± 0.3 kDa

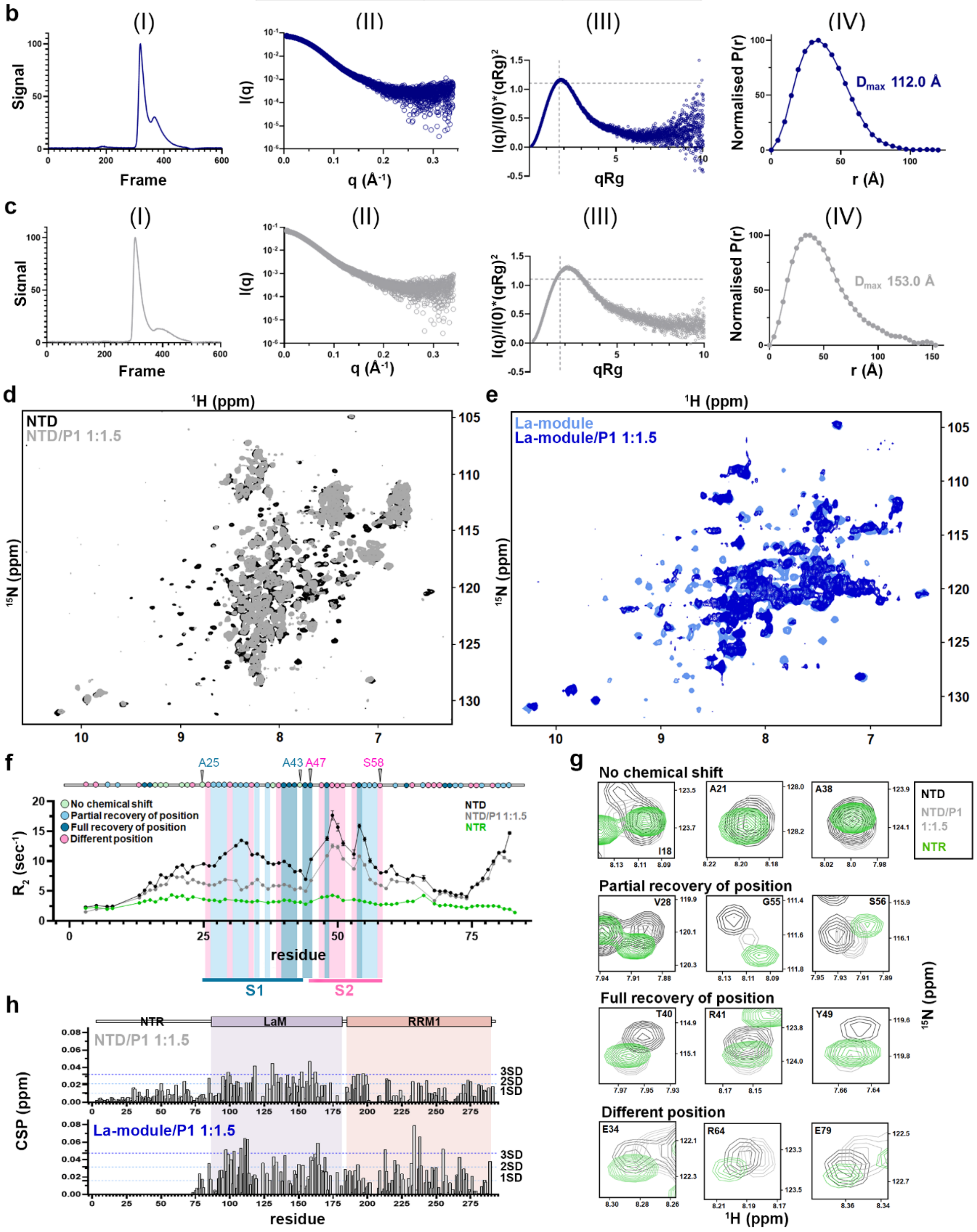

Supplementary Figure 6: Biophysical characterisation of LARP6 La-module and NTD segments in the RNA-bound state.

a) SEC-MALS plots of La-module (left) and NTD (middle) in their apo and RNA bound states. The right panel shows the plot of the free CTNNA1 P1 RNA. The table reports the comparison between observed and expected molecular weights, based on theoretical masses calculation in Expasy ProtParam. The slightly higher masses obtained are consistent with the non-globular shape of the protein. The observed molecular weights of the complexes are compatible with a protein-RNA binding stoichiometry of 1:1.

b) SEC-SAXS characterisation of LARP6 La-module in complex with P1 RNA. The normalised signal of the integral ratio to the background for each frame of SEC-SAXS (I), the X-ray scattering curve obtained after buffer normalisation and averaging (II), the dimensionless Kratky plot (III), and the P(r) distance distribution plot (IV) are depicted.

c) SEC-SAXS characterisation of LARP6 NTD in complex with P1 RNA. The normalised signal of the integral ratio to the background for each frame of SEC-SAXS (I), the X-ray scattering curve obtained after buffer normalisation and averaging (II), the dimensionless Kratky plot (III), and the P(r) distance distribution plot (IV) from the analysis are depicted.

d) Overlay of the full ^{1}H-^{15}N TROSY NMR spectra of LARP6 NTD in the apo state and upon addition of the indicated molar ratios of P1 RNA.

e) Overlay of the full ^{1}H-^{15}N TROSY NMR spectra of LARP6 La-module in the apo state and upon addition of the indicated molar ratios of P1 RNA.

f) Qualitative analysis of NTR chemical shift perturbations in relation to R_2 relaxation rates. Anlysis of the ^{1}H-^{15}N TROSY NMR spectra of the LARP6 NTD in its apo and CTNNA1 P1 RNA-bound states, along with the isolated NTR. Based on the pattern of chemical shift perturbations, NTR residues were classified into four categories: (i) residues showing no perturbation across all three spectra (green); (ii) residues showing partial recovery of their isolated NTR chemical shifts upon RNA binding (light blue); (iii) residues showing full recovery of their isolated NTR chemical shifts upon RNA binding (blue); and (iv) residues adopting distinct chemical shifts when the NTR is tethered to the La-module or when the NTD is RNA-bound (light magenta). In segment S1, most residues exhibited partial or full recovery of their isolated-state chemical shifts upon RNA binding, consistent withh the idea that this region is displaced from the La-module upon RNA engagement. In contrast, residues within segment S2 predominantly showed distinct chemical shifts between the apo and RNA-bound states, suggesting that this region might directly participate in RNA binding. Relaxation rates error bars indicate mean ± SD, estimated from n =100 cycles of Monte Carlo simulation and refitting.

g) Representative peaks belonging to the four categories described in panel (f).

h) Per-residue chemical shift perturbations (CSP) of the NTD residues with or without P1 RNA (top), and the La-module with or without P1 RNA (bottom). The La-module exhibits a higher coverage and larger magnitute of shifts compared to the NTD upon RNA binding (SD: standard deviation).

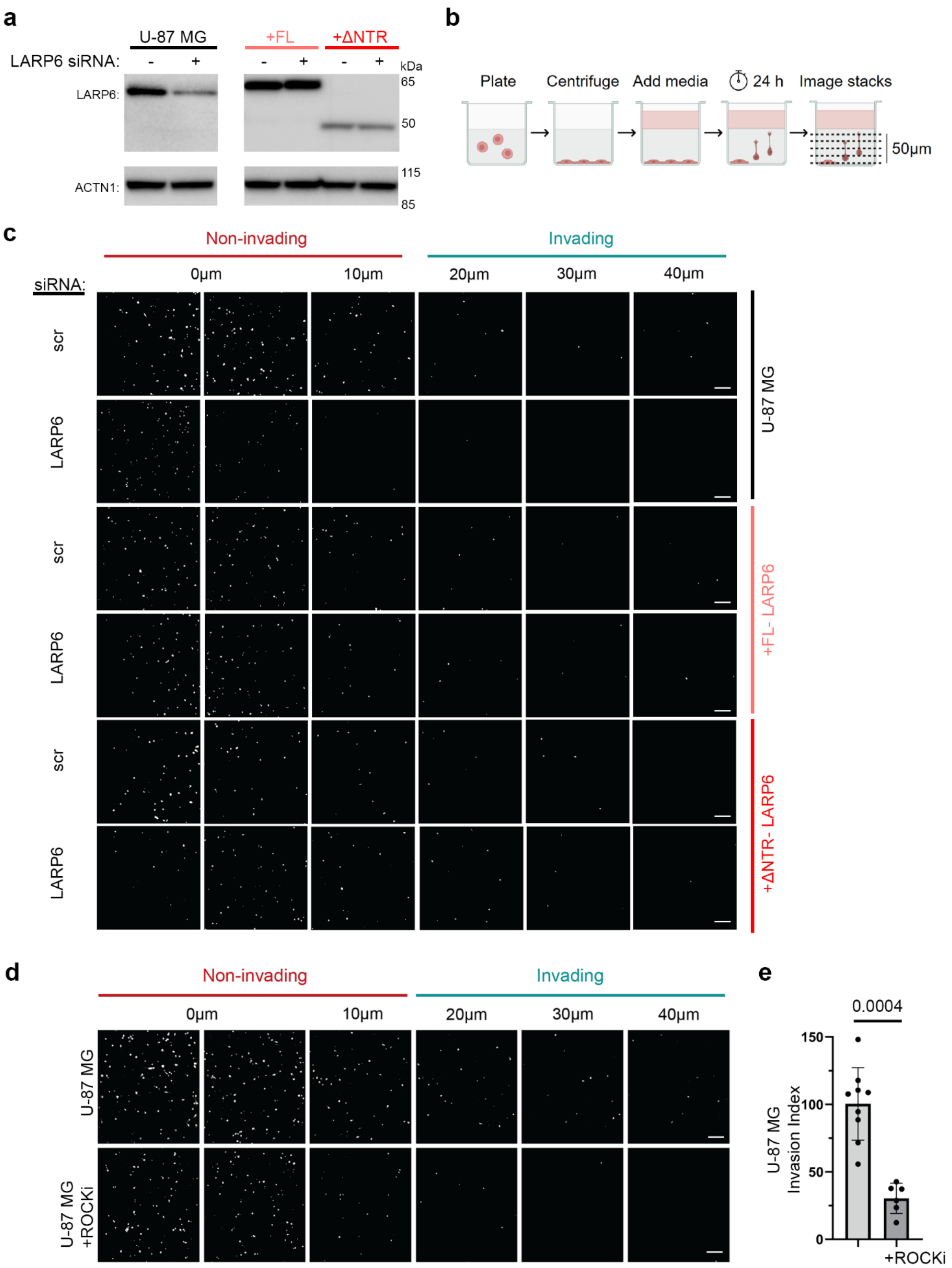

Supplementary Figure 7: Knockdown-rescue analysis of cell invasion by LARP6.

a) Western blot analysis of endogenous LARP6 knockdown in parental U-87 MG cells, and rescue with the indicated myc-LARP6 variants. Note: one representative experiment out of three independent repeats is shown.

b) Experimental workflow of 3D collagen invasion assay. Cells are embedded at the bottom of a collagen-I matrix mixture before allowing it to set into gel. The cells are then induced to invade upwards into the collagen-I matrix for 24 hours by the addition of serum-containing media on the top of the gel, before fixation and analysis of the number of invaded cells by confocal microscopy.

c) Representative images of the invasion assay from Figure 5c. Nuclei are shown in white and were used at each z-plane to count the number of non-invaded vs. invaded cells. Scale bars = 100 μm.

d) Representative images of invasion assay in parental cells with or without 1μM AT13148 Rho kinase inhibitor (ROCKi). Nuclei are shown in white and were used at each z-plane to count the number of non-invaded vs. invaded cells. Scale bars = 100 μm.

e) Invasion index quantification from (d). The invasion index is normalised to the untreated control samples without ROCKi. Error bars = mean ± SD (n = 9 (Ctrl) or 6 (treated) biological replicates). A two sided Kolmogorov–Smirnov test was applied for significance calculation. The *p*-value is indicated on the bar graph.

Supplementary References:

1. Cowieson, N.P. et al. Beamline B21: high-throughput small-angle X-ray scattering at Diamond Light Source. *J Synchrotron Radiat* **27**, 1438-1446 (2020).
2. Nusinow, D.P. et al. Quantitative Proteomics of the Cancer Cell Line Encyclopedia. *Cell* **180**, 387-402 e16 (2020).
3. Marsh, J.A., Singh, V.K., Jia, Z. & Forman-Kay, J.D. Sensitivity of secondary structure propensities to sequence differences between alpha- and gamma-synuclein: implications for fibrillation. *Protein Sci* **15**, 2795-804 (2006).

Uncropped Blot Scans:

Figure S1a:

Anti-GFP (1 min exposure):

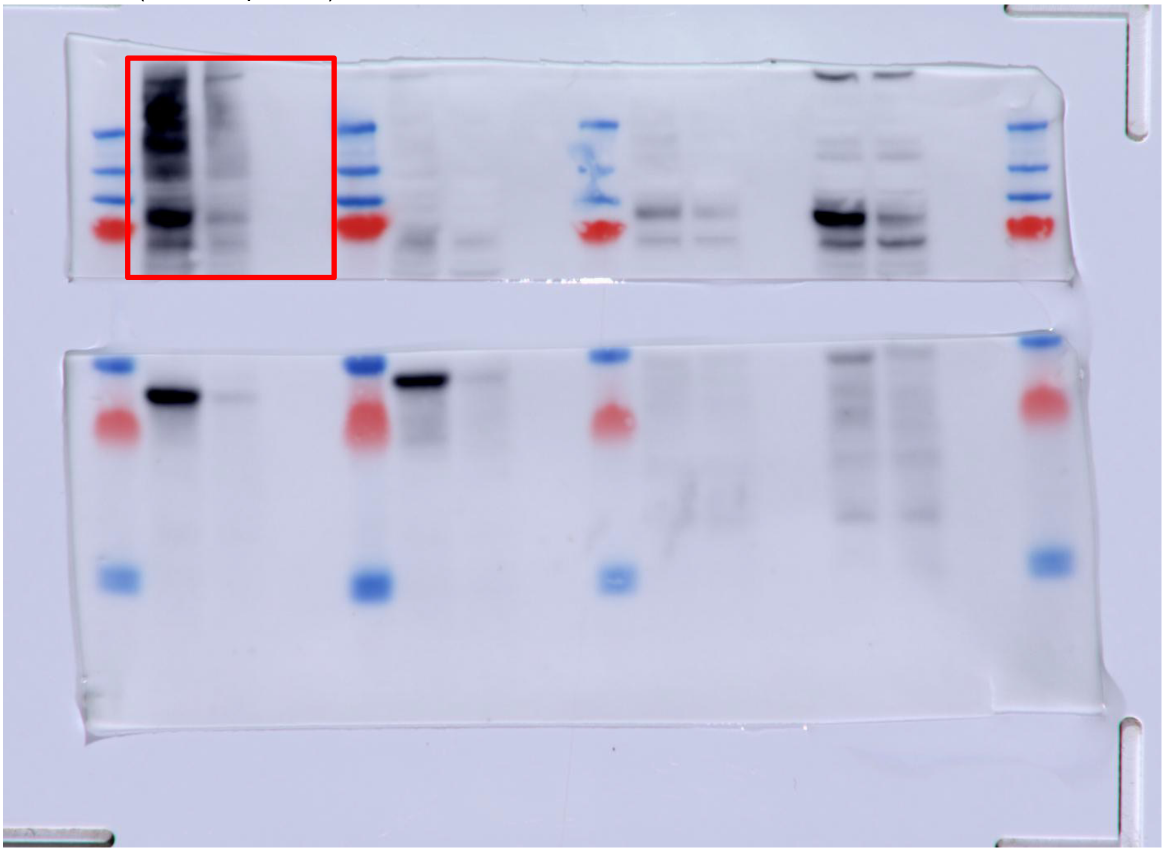

GAPDH (7 sec exposure):

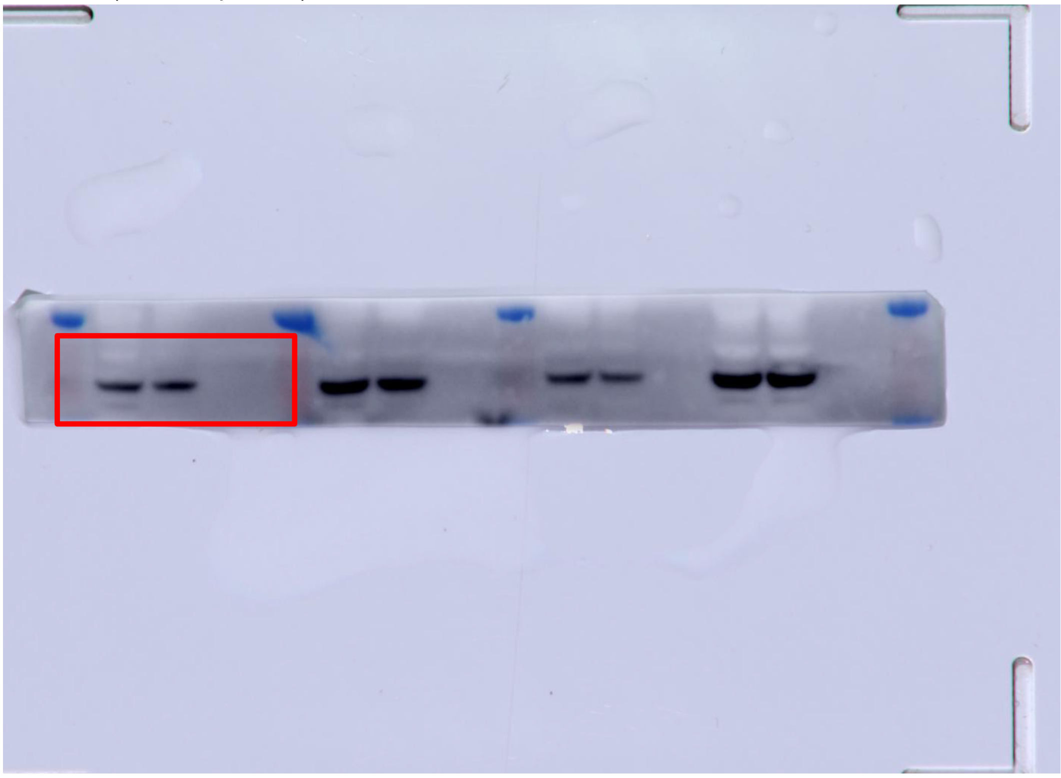

Figure S2c:

LARP6 (170 sec exposure):

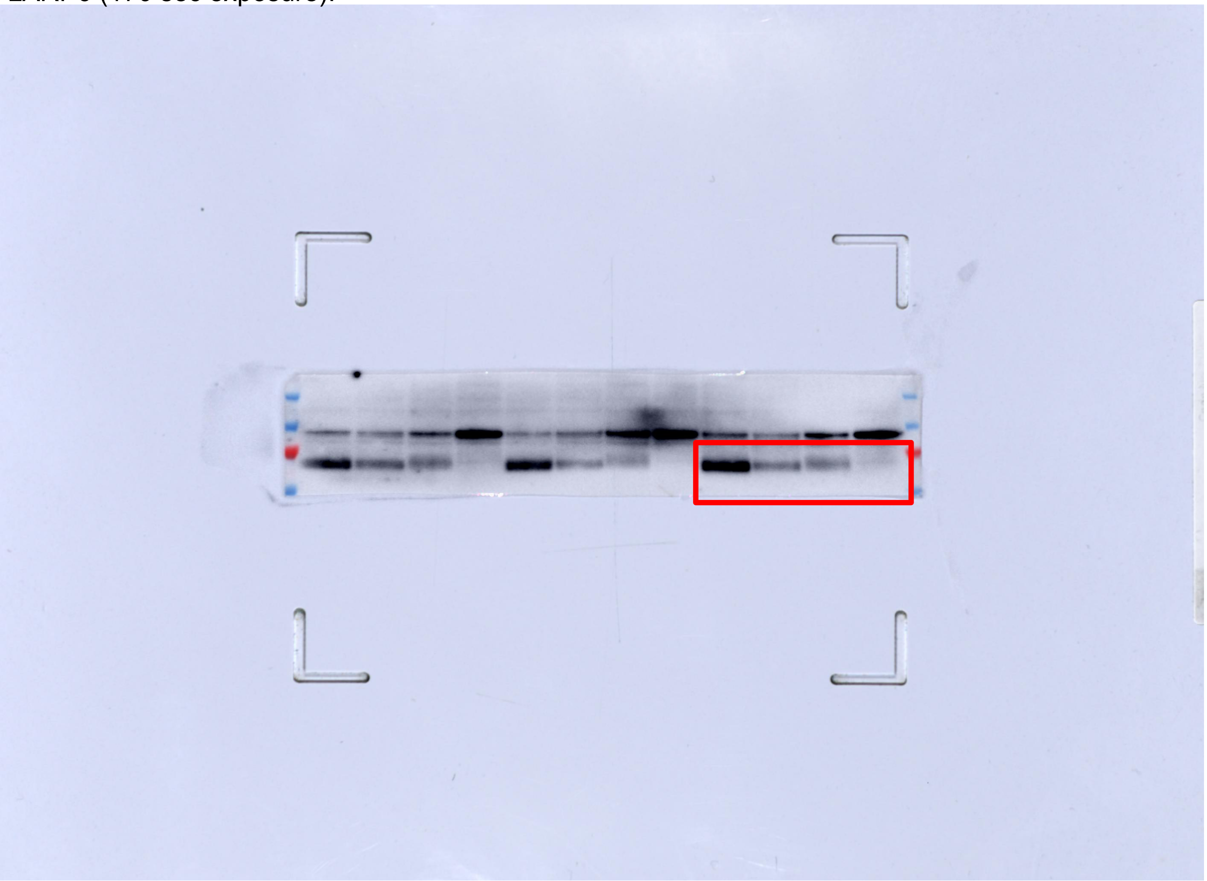

GAPDH (120 sec exposure):

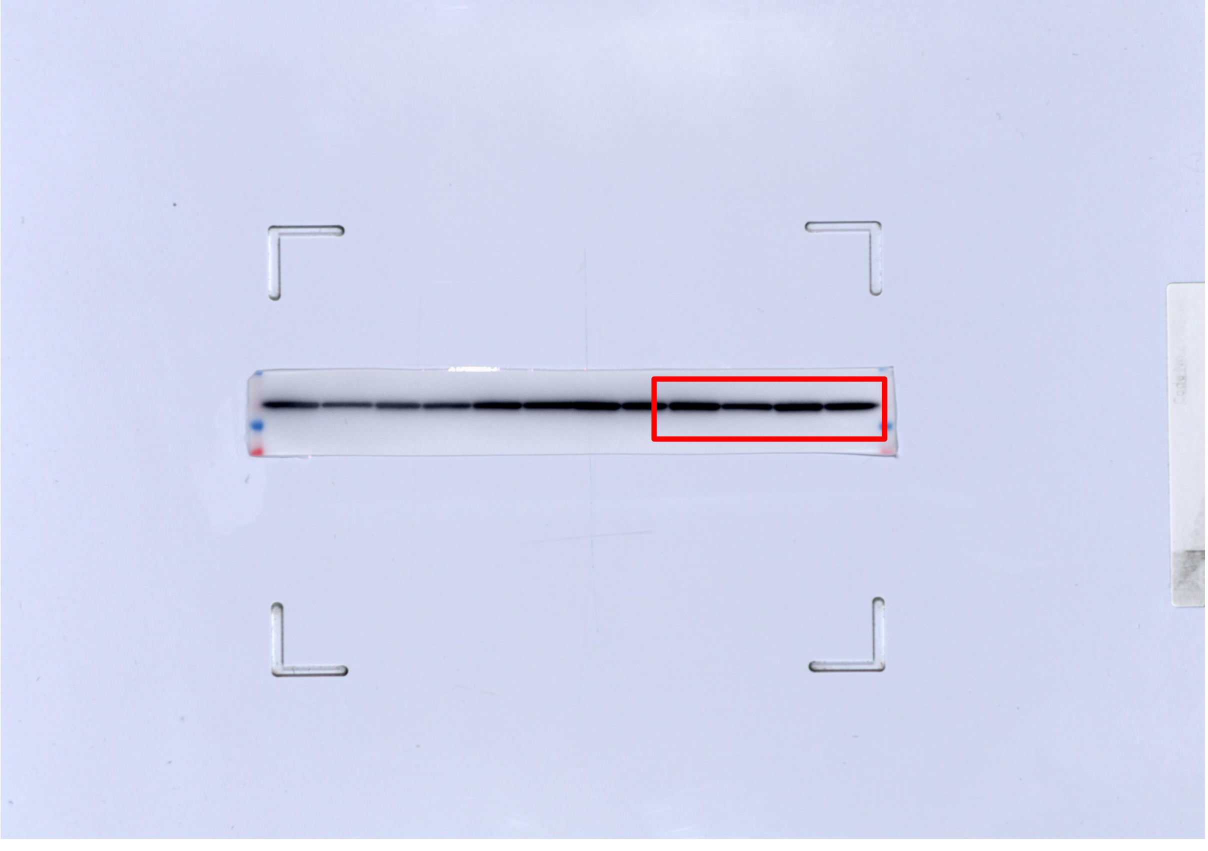

Figure S2e:

LARP6 (20 sec exposure):

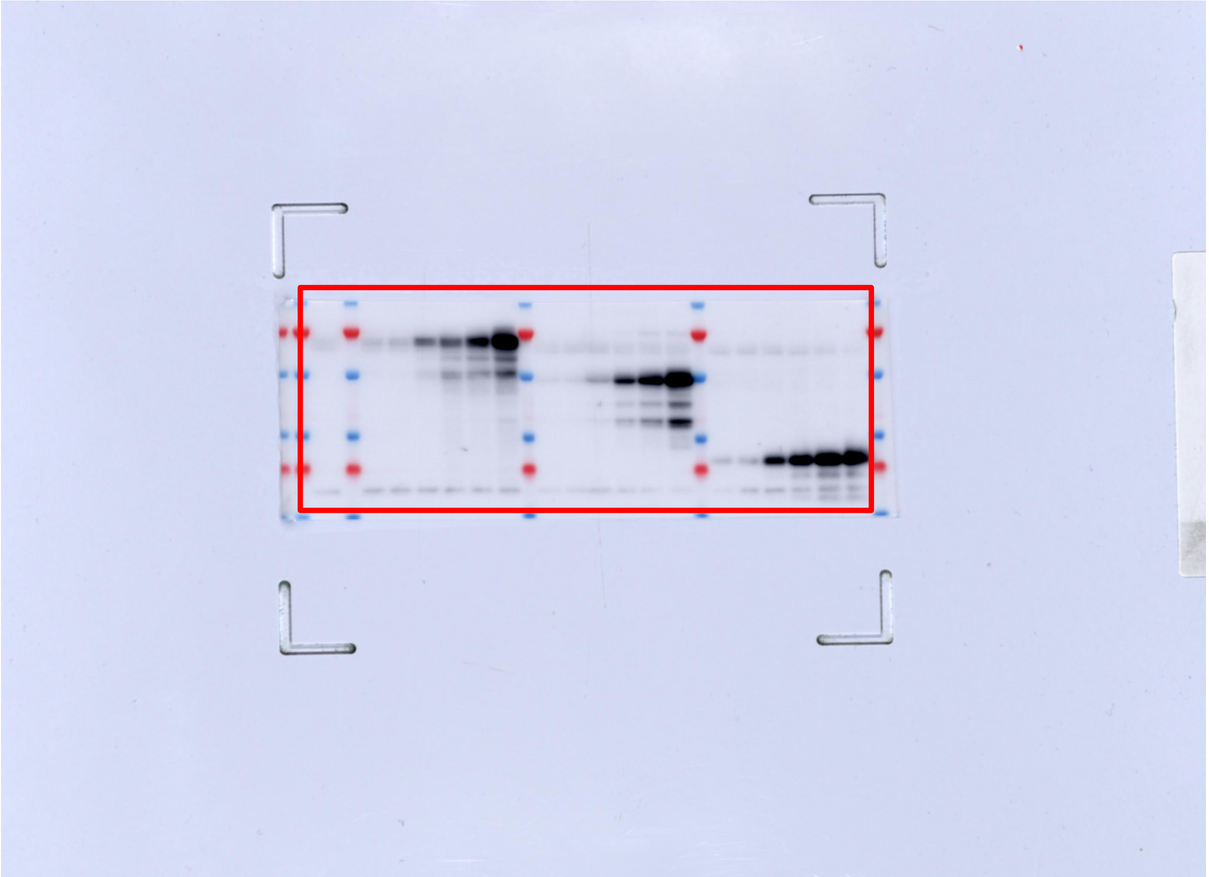

Actinin (30 sec exposure):

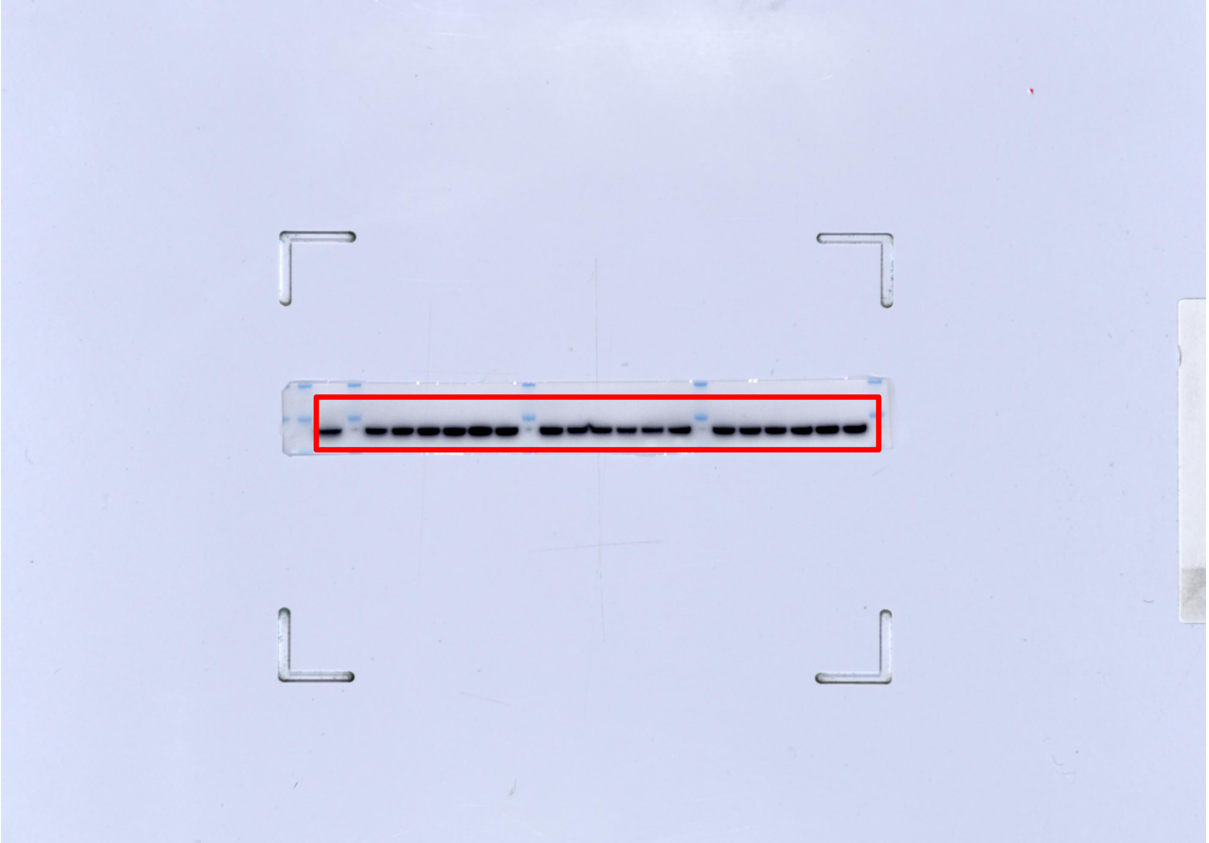

Figure S2f:

LARP6 (40 sec exposure):

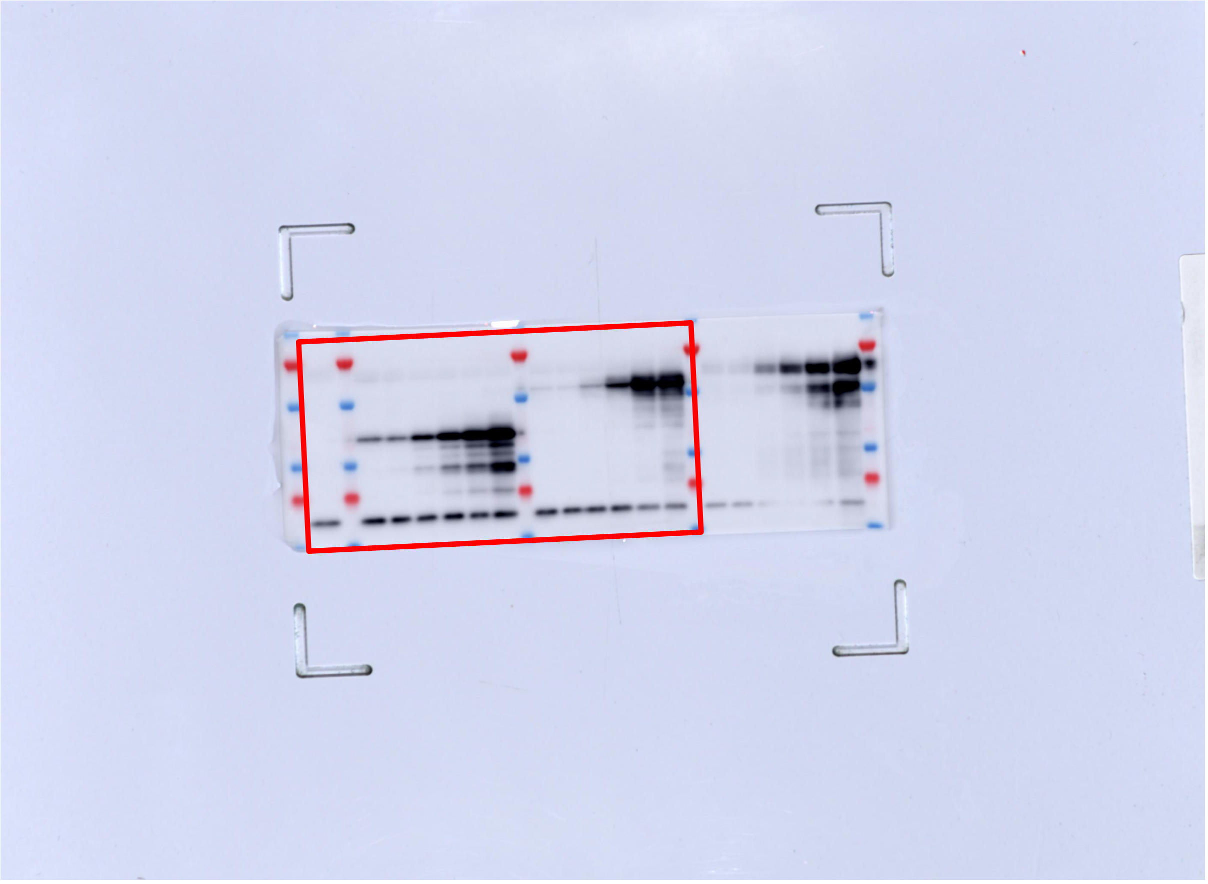

Actinin (34 sec exposure):

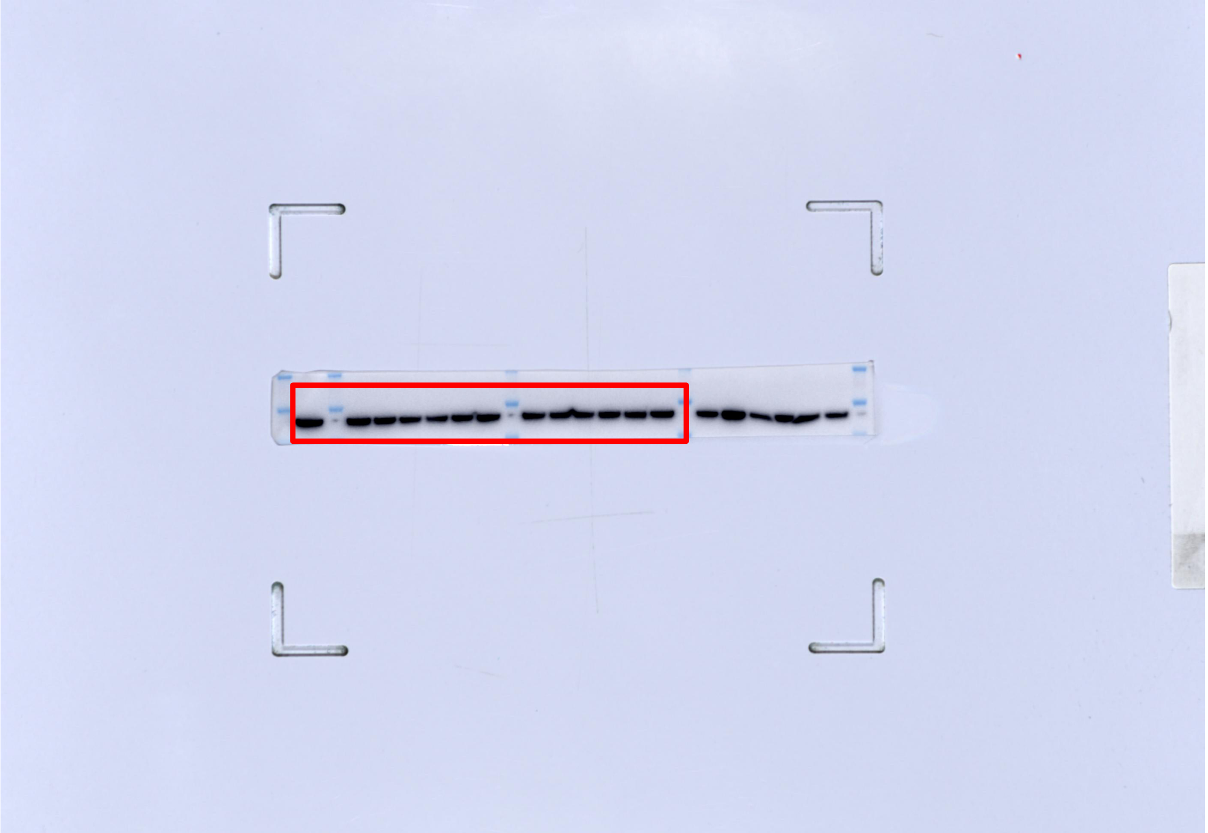

Figure S7a:

LARP6 (4 sec exposure):

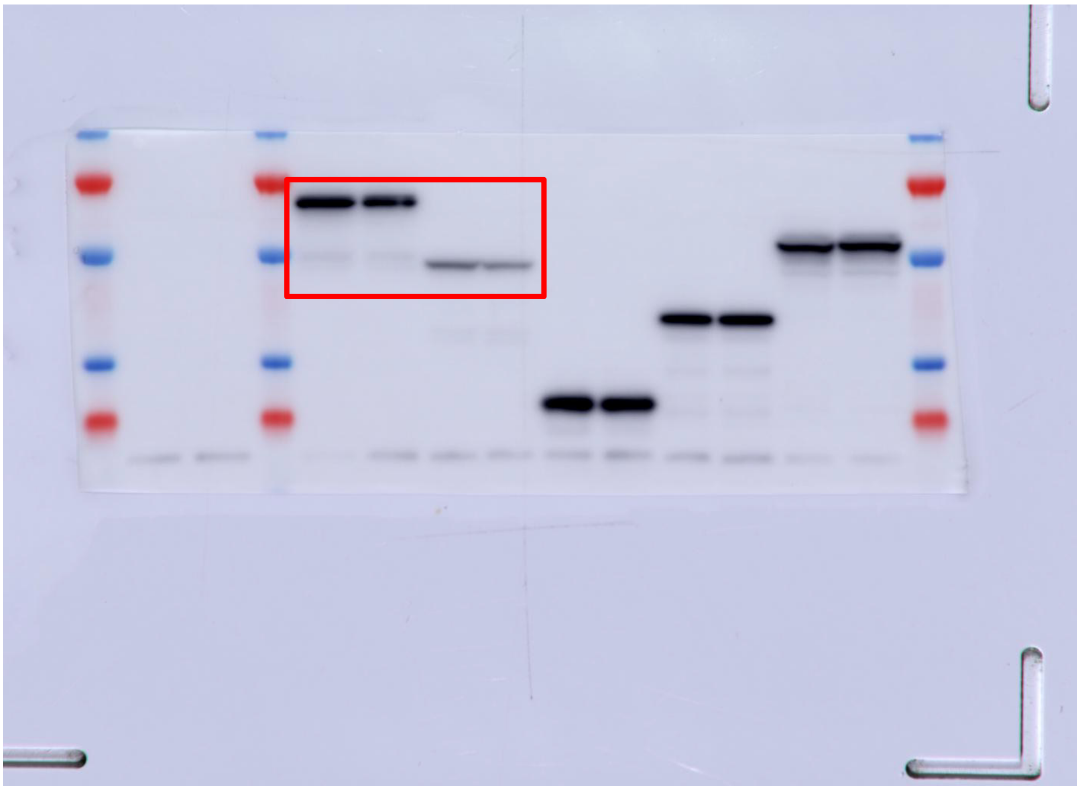

LARP6, parental only (4 sec exposure):

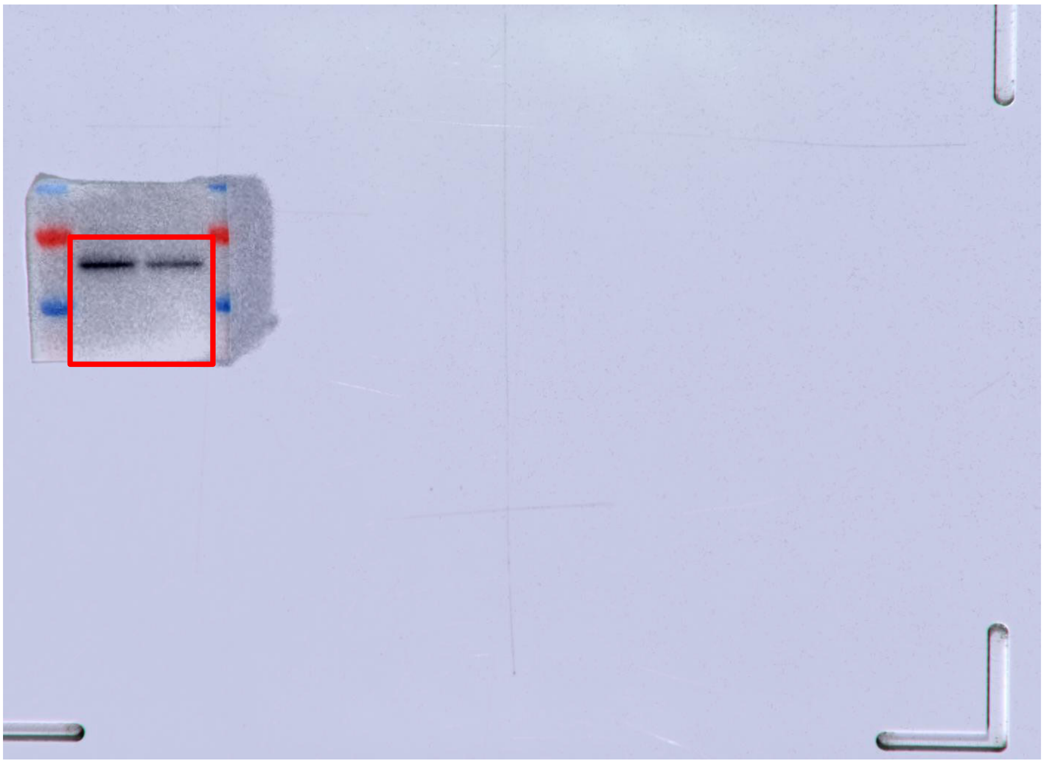

Actinin (10 sec exposure):

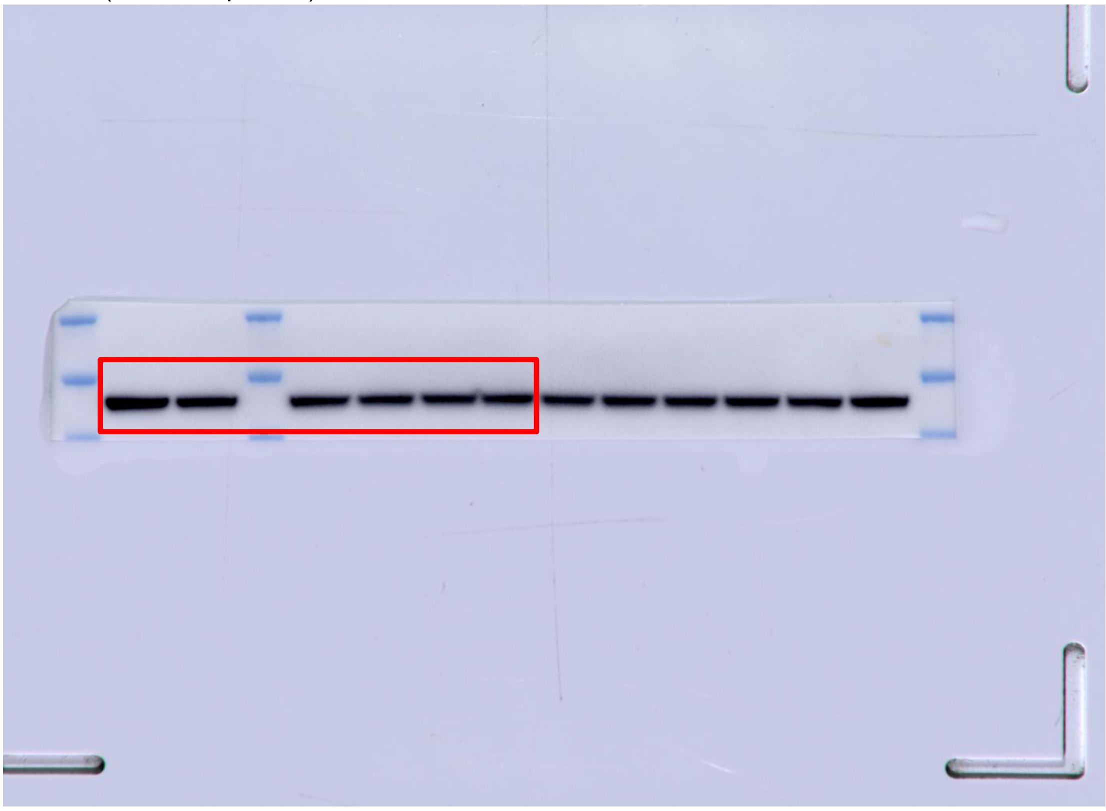

Supplement: Supplementary file 1 — Supplementary Information [file 41467_2026_69789_MOESM1_ESM.pdf]
