# Supplementary material for: An intrinsically disordered region mediates RNA-binding selectivity and cellular activities of LARP6
Source: Nat Commun. 2026 Feb 19;17:2939. doi: 10.1038/s41467-026-69789-z (PMC13031558; doi:10.1038/s41467-026-69789-z)
Supplement: Supplementary file 2 — Description of Additional Supplementary Files [file 41467_2026_69789_MOESM2_ESM.pdf]

Description of Additional Supplementary Files

Supplementary Data 1: List of FL myc-LARP6 iCLIP peaks as per genomic coordinates, and the associated peak scores.

Supplementary Data 2: List of ΔNTR myc-LARP6 iCLIP peaks as per genomic coordinates, and the associated scores.

Supplementary Data 3: List of ΔCTR myc-LARP6 iCLIP peaks as per genomic coordinates, and the associated scores.

Supplementary Data 4: Transcriptome-wide RNA-seq analysis of Protrusion/cell-body mRNA distributions in U87 Glioblastoma cells, with or without LARP6 depletion, and rescue with FL or ΔNTR myc-LARP6.
