# Supplementary material for: An intrinsically disordered region mediates RNA-binding selectivity and cellular activities of LARP6
Source: Nat Commun. 2026 Feb 19;17:2939. doi: 10.1038/s41467-026-69789-z (PMC13031558; doi:10.1038/s41467-026-69789-z)
Supplement: Supplementary file 7 — Reporting Summary [file 41467_2026_69789_MOESM7_ESM.pdf]

nature portfolio

Corresponding author(s): Faraz K. Mardakheh
Department of Biochemistry
University of Oxford
South Parks Road
Oxford OX1 3QU
The United Kingdom

Last updated by author(s): Feb 6, 2026

Reporting Summary

Nature Portfolio wishes to improve the reproducibility of the work that we publish. This form provides structure for consistency and transparency in reporting. For further information on Nature Portfolio policies, see our Editorial Policies and the Editorial Policy Checklist.

Statistics

For all statistical analyses, confirm that the following items are present in the figure legend, table legend, main text, or Methods section.

n/a	Confirmed	
☐	☒	The exact sample size (n) for each experimental group/condition, given as a discrete number and unit of measurement
☐	☒	A statement on whether measurements were taken from distinct samples or whether the same sample was measured repeatedly
☐	☒	The statistical test(s) used AND whether they are one- or two-sided<br>*Only common tests should be described solely by name; describe more complex techniques in the Methods section.*
☐	☒	A description of all covariates tested
☐	☒	A description of any assumptions or corrections, such as tests of normality and adjustment for multiple comparisons
☐	☒	A full description of the statistical parameters including central tendency (e.g. means) or other basic estimates (e.g. regression coefficient) AND variation (e.g. standard deviation) or associated estimates of uncertainty (e.g. confidence intervals)
☐	☒	For null hypothesis testing, the test statistic (e.g. F, t, r) with confidence intervals, effect sizes, degrees of freedom and P value noted<br>*Give P values as exact values whenever suitable.*
☒	☐	For Bayesian analysis, information on the choice of priors and Markov chain Monte Carlo settings
☒	☐	For hierarchical and complex designs, identification of the appropriate level for tests and full reporting of outcomes
☒	☐	Estimates of effect sizes (e.g. Cohen's d, Pearson's r), indicating how they were calculated

Our web collection on statistics for biologists contains articles on many of the points above.

Software and code

Policy information about availability of computer code

Data collection	*Provide a description of all commercial, open source and custom code used to collect the data in this study, specifying the version used OR state that no software was used.*
Data analysis	Maxquant (version 1.6.6.3); Perseus (version 1.6.2.1); iMAPS webserver (imaps.goodwright.com); Ultraplex (version 1.1.4); TrimGalore!; Bowtie 2; UMItools (versions 1.1.1 & 1.1.4); Trim-galore (version 0.6.5); STAR (versions 2.6.1a); Clippy (version 1.5.0); bedtools (version 2.30.0); ComBat; metagene2 (version 1.18.0); iCount; Cutadapt (version 1.18); Pythia (https://github.com/goodarzilab/pythia); Octed BLI Discovery software (12.2.2.20); Octet BLI Analysis software (12.2.2.4); IUPred2A webserver (iupred2a.elte.hu); ScÅtter IV; IGV-Web (version 1.13.9); ASTRA One-Click MW; ExPASy ProtParam tool; PyMOL Molecular Graphics System (version 2.0); Microsoft Office 365; GraphPad Prism (versions 7 or 10); NMRPipe; TopSpin® Bruker; CcpNmr Analysis (Version 3); SSP package (https://pound.med.utoronto.ca/JFKlab/index.php#SSP); MO.Affinity Analysis (version 2.2.4) (NanoTemper); iCLIP annotation script (custom-code: https://github.com/alejrb/iclip-annotation/);

For manuscripts utilizing custom algorithms or software that are central to the research but not yet described in published literature, software must be made available to editors and reviewers. We strongly encourage code deposition in a community repository (e.g. GitHub). See the Nature Portfolio guidelines for submitting code & software for further information.

Data

Policy information about availability of data

All manuscripts must include a data availability statement. This statement should provide the following information, where applicable:

- Accession codes, unique identifiers, or web links for publicly available datasets
- A description of any restrictions on data availability
- For clinical datasets or third party data, please ensure that the statement adheres to our policy

All mass spectrometry and NGS sequencing datasets were deposited onto public repositories. Specifically PRIDE was used for mass spectrometry data, and GEO was used for next-generation RNA-sequencing data. The datasets have been made publicly available. All NMR assignments were submitted to the Biological Magnetic Resonance Bank (BMRB), under the entry number 53427. All SAXS data were submitted to the SasBDB80, under the entry number SASDY26.

Research involving human participants, their data, or biological material

Policy information about studies with human participants or human data. See also policy information about sex, gender (identity/presentation), and sexual orientation and race, ethnicity and racism.

Reporting on sex and gender	Not relevant to this study
Reporting on race, ethnicity, or other socially relevant groupings	Not relevant to this study
Population characteristics	Not relevant to this study
Recruitment	Not relevant to this study
Ethics oversight	Not relevant to this study

Note that full information on the approval of the study protocol must also be provided in the manuscript.

Field-specific reporting

Please select the one below that is the best fit for your research. If you are not sure, read the appropriate sections before making your selection.

[x] Life sciences [ ] Behavioural & social sciences [ ] Ecological, evolutionary & environmental sciences

For a reference copy of the document with all sections, see nature.com/documents/nr-reporting-summary-flat.pdf

Life sciences study design

All studies must disclose on these points even when the disclosure is negative.

Sample size	Pre-determination of sample size was not possible in this study. We always included at least 3 biological replicates for all the presented work.
Data exclusions	No data was excluded from any of the analyses
Replication	High-throughput experiments (e.g. RNA-seq and iCLIP) were performed in triplicates, while functional analyses were often repeated more. In all graphs, individual datapoints have always been plotted to depict the number of repeats, and this information is also stated in figure legends.
Randomization	Not relevant to this work
Blinding	Not relevant to this work

Reporting for specific materials, systems and methods

We require information from authors about some types of materials, experimental systems and methods used in many studies. Here, indicate whether each material, system or method listed is relevant to your study. If you are not sure if a list item applies to your research, read the appropriate section before selecting a response.

Materials & experimental systems

n/a	Involved in the study
☐	☒ Antibodies
☐	☒ Eukaryotic cell lines
☒	☐ Palaeontology and archaeology
☒	☐ Animals and other organisms
☒	☐ Clinical data
☒	☐ Dual use research of concern
☒	☐ Plants

Methods

n/a	Involved in the study
☒	☐ ChIP-seq
☒	☐ Flow cytometry
☒	☐ MRI-based neuroimaging

Antibodies

Antibodies used

Primary Abs.:
1- Rabbit anti-LARP6 (polyclonal), 1:200 (WB), 1:58 (IF), Atlas Antibodies, HPA049029 (lot 000022824).
2- Mouse anti-myc-tag (9B11), 1:1000 (WB), 1:500 (IF), Cell Signaling, 2276S.
3- Mouse anti-GAPDH (1D4), 1:500 (WB), Novus Biologicals, NB300-221.
4- Mouse anti-GFP (B-2), 1:1000, Santa Cruz, sc-9996.
Secondary Abs.:
1- Amersham ECL peroxidase labelled anti-rabbit, 1:5000 (WB),Cytiva, NA934VS.
2- Amersham ECL peroxidase labelled anti-mouse, 1:5000 (WB), Cytiva, NA931VS.
3- Alexa Fluor 594 AffiniPure donkey anti-rabbit IgG, 1:400 (IF), Jackson, 711-515-152.
4- Fluorescein (FITC) AffiniPure donkey anti-mouse IgG, 1:400 (IF), Jackson, 715-095-151.

Validation

The specific batch of LARP6 polyclonal antibody was validated by siRNA knockdown of endogenous LARP6 and performing WB or IF. All other antibodies (e.g. primary and secondary) were validated by the manufacturer and extensively used in publications.

Eukaryotic cell lines

Policy information about cell lines and Sex and Gender in Research

Cell line source(s)	U-87 MG (ATCC – cat no. HTB-14); HEK293-T (source: Prof. Chris Marshall's lab, Institute of Cancer Research, London)
Authentication	STR profiling
Mycoplasma contamination	Cells were regulary tested for mycoplasma and were always free of contamination
Commonly misidentified lines (See ICLAC register)	none

Plants

Seed stocks	N/A
Novel plant genotypes	N/A
Authentication	N/A
